# Induced Pluripotent Stem Cells: Development in the Ophthalmologic Field

**DOI:** 10.1155/2016/2361763

**Published:** 2016-08-10

**Authors:** Nan Wu, Marianne Doorenbos, Dong Feng Chen

**Affiliations:** ^1^Department of Ophthalmology, Southwest Eye Hospital, Southwest Hospital, Third Military Medical University, Chongqing 400038, China; ^2^Schepens Eye Research Institute, Massachusetts Eye and Ear, Department of Ophthalmology, Harvard Medical School, Boston, MA 02114, USA; ^3^Department of Ophthalmology, University of Groningen, University Medical Center Groningen, 9713 GZ Groningen, Netherlands

## Abstract

Human induced pluripotent stem cells (iPSCs) are a type of stem cells that can be derived from human somatic cells by introducing certain transcription factors. Induced pluripotent stem cells can divide indefinitely and are able to differentiate into every cell type, which make them viable for transplantation and individual disease modeling. Recently, various ocular cells, including corneal epithelial-like cells, retinal pigment epithelium (RPE) cells displaying functions similar to native RPE, photoreceptors, and retinal ganglion cells, have all been successfully derived from iPSCs. Transplantation of these cells in animal models showed great promise for reversing blindness, and the first clinical trial on humans started in 2013. Despite these promising results, more research is in demand for preventing inadvertent tumor growth, developing precise functionality of the cells, and promoting integration into the host tissue.

## 1. Introduction: Potential of Stem Cell Therapy for Untreatable Ocular Diseases

Retinal degenerative diseases, such as age-related macular degeneration, retinitis pigmentosa, and glaucoma, are major causes of irreversible blindness worldwide. The retina is a complex multilayered neural tissue that converts light energy to electrical signals, which are relayed through the optic nerve to the occipital lobe of the brain to undergo visual processing. To date, damage to, or degeneration of, any part of the retina is permanent. Currently there is increasing interest in repairing damaged tissues with pluripotent stem cells which can divide indefinitely and have the potential to generate multiple types of cells. These characteristics of stem cells offer the opportunity to repair virtually all types of tissues, including the retina, through cell replacement or transplantation or to model the disease process.

**Figure 1:**
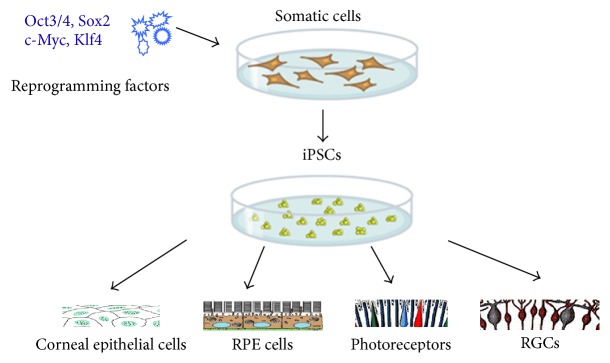
Schematic illustration of iPSC induction and reprogramming into ocular cells. iPS cells are generated by reprogramming adult somatic cells. In the ophthalmic field, iPSCs have been successfully differentiated into a variety of the ocular cells, including corneal epithelial cells, RPE, photoreceptors, and RGCs.

Human embryonic stem cells (hESCs) are the first characterized pluripotent stem cells that could be induced to generate all types of cells including retinal neurons, for example, retinal pigment epithelium (RPE) cells [1, 2]. Another viable alternative is to induce stem cells from autologous somatic cells. Built on top of John Gurdon's earlier work in* Xenopus*, Takahashi and Yamanaka showed that pluripotent stem cells could be generated from mouse fibroblast cultures by adding four transcription factors: Oct3/4, Sox2, c-Myc, and Klf4 [3]. In 2012, Yamanaka and Gurdon won the Nobel Prize for their combined efforts in reprogramming mature cells into embryonic cells (Figure 1). As iPSCs can be derived from patient's own somatic cells, it eliminates the problems of posttransplantation rejection and ethical concerns surrounding the use of embryonic cells. These cells also offer an opportunity to access patient's genetics and allow identification of the disease-initiating events. Nowadays, the technology has been applied to investigate the physiology, disease pathogenesis, and therapeutic potential of drugs through tissue modeling, which includes cardiomyocytes [4], hepatocytes [5], and bone and cartilage cells [6]. iPSCs thus are of promise for both novel patient-specific treatments and disease modeling.

Application of iPSCs or the derived cells to disease therapy depends on the studies to understand the integrity of the iPSCs, their potential for tumor formation, immunogenicity, and epigenetic aberrations which may occur during the reprogramming process. Miura et al. reported that the type of tissue from which iPSCs are originated is the most important determinant for teratoma formation after transplantation in brains of immunodeficient mice [7]. When iPSCs were transplanted into murine models of contusional spinal cord trauma, iPSC clones originated from mouse embryonic fibroblasts were considered safe, while those from adult tail-tip fibroblasts were unsafe due to severe teratoma formation [8]. To date, despite the safety and integrity concerns, studies demonstrating the treatment efficacy in various disease models have made the further research into iPSCs very interesting and worthwhile.

In the ophthalmic field, iPSCs or derivatives of iPSCs present a promising treatment modality. Degenerative diseases, such as age-related macular degeneration and glaucoma, are common but incurable. Utilization of iPSCs as a low immunogenic and patient-specific source for stem cells to replace damaged or diseased ocular cells, including corneal epithelial cells, RPE, photoreceptors, and RGCs, could be an excellent way to restore visual function in otherwise untreatable conditions (Figure 1). In this review, we describe recent developments of stem cell therapy in the ocular field, particularly the progress made in using iPSCs toward the treatment for corneal dystrophy, age-related macular degeneration (RPE or photoreceptors), and optic nerve (RGCs) diseases.

## 2. iPSCs in Ocular Therapy: Deriving Corneal Epithelial-Like Cells from iPSCs

A healthy and transparent cornea is a prerequisite for visual activity, so corneal ulceration and scarring have been a matter of interest in the ocular field. Although superficial corneal abrasions are self-limited [9, 10], complications like corneal ulcers and scarring can be difficult to treat. The idea of treating injuries of the ocular surface with derivatives of iPSCs is appealing.

The ocular surface is made up of corneal and conjunctival epithelial cells. Limbal cells have been identified as a source of adult stem cells [11], which enable corneal wound healing in a centripetal way [12]. In patients suffering chemical or thermal injury to the corneal epithelium in one eye, autologous transplantation of limbal cells taken from the healthy eye allows epithelium cytologically consistent with corneal epithelium [13]. Because autologous transplantation requires donor tissue from the healthy eye, which is not possible in patients with bilateral injury, Pellegrini et al. searched for an alternative by culturing limbal cells from full-thickness biopsy [14]. They were able to grow a sheet of corneal epithelial cells from the biopsy, which was then transplanted successfully into the patient's injured eye and resulted in significant improvement of visual acuity.

**Figure 2:**
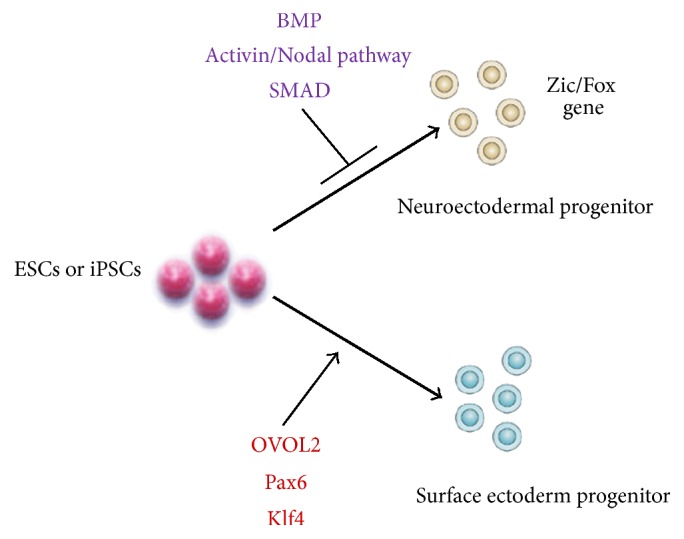
Schematic illustration of corneal epithelial cell differentiation from ESCs or iPSCs. Cultured human iPSCs treated with competitors of BMP, Activin, and Nodal pathways that result in the inhibition of Smad signaling become neuroectodermal progenitors due to activation of earlier neuronal genes (i.e., Zic and Fox family members). In contrast, OVOL2, repressed mesenchymal genes, together with PAX6 and Klf4, activate corneal epithelium differentiation. BMP, bone morphogenetic proteins; OVOL2, ovo-like zinc finger 2; Pax6, paired box protein Pax-6; Klf4, Kruppel-like factor 4; Zic, zinc finger; Fox, forkhead/winged-helix.

Although patient's own limbal stem cells have proven to be a successful source for transplantation therapy to treat corneal epithelium deficiency, the need for an alternative source of stem cells is apparent as bilateral disease can only be treated with allogeneic grafts. Homma et al. thus studied growth and transplantation of ESC-derived epithelial progenitors into the injured cornea [15]. Corneal epithelium-like cells were first differentiated from human iPSCs in 2012 [16]. A more efficient method, in which two small-molecule inhibitors were applied to stimulate the early development of iPSC-derived cells, was recently developed by Mikhailova et al. [17]. Modulation of intracellular signaling pathways could generate neuroectoderm lineage or desirable cell types and partly overcome innate differentiation propensity.  Figure 2 recapitulates the process used to differentiate ESCs/iPSCs toward the neuroectoderm [18] or surface ectoderm (corneal epithelium). Although no clinical trials have yet been performed, these studies showed great promise for autologous treatment of corneal disease with iPSC-derived cells that enables elimination of the use of immunosuppressive therapies necessary for allogeneic grafts.

## 3. iPSC-Derived RPE for Photoreceptor Degeneration Therapy

Retinal degeneration is a serious disease that occurs in patients of all ages, such as Stargardt's disease in younger patients and age-related macular degeneration in the eldery. Despite the differences in time of onset, any damage to the retina is untreatable and irreversible to this day. The idea of repairing the degenerated cells with “fresh” iPSC-derived cells has been very appealing.

The retina is a multilayered tissue that lies posteriorly in the eye alongside the choroid and is responsible for the blood supply of the outer retina. The rod and cone photoreceptors that absorb and sense the light work closely together with the RPEs. The main functions of the RPE are to transport nutrients and waste products to and from the photoreceptor layer, participate in vitamin A-rhodopsin conversion cycle, phagocyte outer segments of photoreceptors, and absorb scattered light to improve visual acuity. Visual information detected by photoreceptors is conveyed to the brain via retinal ganglion cells (RGCs), which connect to photoreceptors through bipolar cells, whose nuclei reside in the inner nuclear layer. The cell bodies of RGCs from the ganglion cell layer and their axons meet in the optic nerve head and form the optic nerve.

As mentioned above, the RPE layer is crucial to the survival and function of photoreceptors; thus, dysfunction of the RPE can be a disease-initiating event and is thought to associate with age-related macular degeneration [19] and retinitis pigmentosa [20]. The therapeutic potential of RPE cell transplantation has thus been investigated both in animal models and in humans for a long time [21]. Two strategies are being used for RPE cell transplantation, injected as a cell suspension or a monolayer cell sheet. Just a few years ago,highly homogenous RPE cells were generated from iPSCs following procedures similar to those used in the differentiation of RPE cells from ESCs [22]. Buchholz et al. used another set of factors, the factors developed by Jamie Thomson's group, Oct4, Sox2, Nanog, and Lin28, to derive RPE cells from iPSCs [23]. These derived cells were similar to native RPE cells in protein expression, gene expression, and phagocytosis of shedded photoreceptor outer segments. Further studies showed that the membrane potential, ion transport, and secretion of vascular endothelial growth factor in RPE cells derived from iPSCs are all similar to native RPE [24]. However, telomere shortening and rapid senescence were observed after several cell cycles, which lead to impaired function [25]. As suggested by Kokkinaki and colleagues [24], properly functioned RPE cells could be preserved if only cells from the first few passages are used. Through genetic profiling of RPE, the differentiation status of the iPSC-derived RPE can now be determined by molecular markers [26]. Recently, simplified differentiation protocols that enable acquisition of a larger quantity of cells without the use of feeder-cells or xenogenic material have been developed, thereby paving the way for clinical trials [27].

## 4. iPSC Strategy for Replacing Photoreceptor Cells

**Figure 3:**
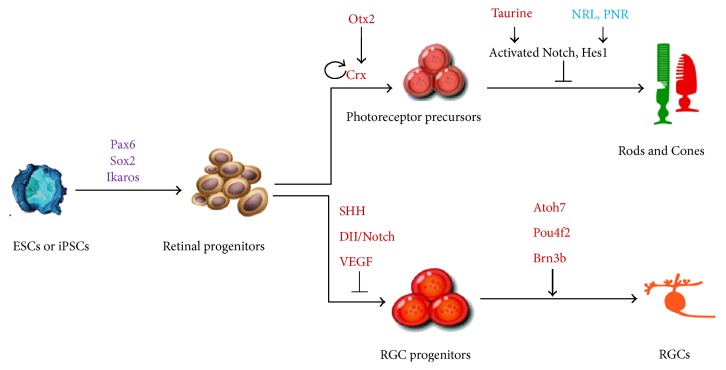
Schematic illustration of photoreceptor (rod and cone) or RGC production from ESCs or iPSCs. Pax6, paired box protein Pax-6; Sox2, SRY- (sex determining region Y-) box 2; Ikaros, Ikaros transcription factor; Otx2, orthodenticle homeobox 2; CRX, cone-rod homeobox; NRL, neural retina leucine zipper gene; PNR, photoreceptor cell-specific nuclear receptor; Hes1, hes family BHLH transcription factor 1; SHH, sonic hedgehog; DII, the drosophila distal-less; VEGF, vascular endothelial growth factor; Atoh7, atonal BHLH transcription factor 7; Pou4f2, POU domain, class 4, transcription factor 2.

When there is extensive loss of photoreceptors with or without RPE involvement, a source of photoreceptor cells will be needed. In contrast to protocols aimed at RPE transplantation, neural retina replacement is far more challenging. This is not only because generation of photoreceptors from iPSCs tends to require greater manipulation of the culture environment, but also because transplanted neurons must also develop appropriate neuronal connectivities with the host. Nowadays, donor precursor photoreceptor cells acquired from developing mouse retinas have been shown to survive, integrate, and improve visual function after subretinal transplantation in both wildtype and degenerative mouse models [28]. Multiple studies reported that human ESCs can be differentiated into photoreceptor and other retinal neuron progenitors, which further differentiate into cells with characteristics similar to naïve photoreceptors and RGCs (Figure 3) [29–31]. Animal studies with subretinal transplantation of human ESC-derived photoreceptors, however, obtained mixed results. West and colleagues experienced tumor formation after the transplantation of early stage retinal progenitors but observed no robust integration into the retina when later stage photoreceptors were used [30]. Lamba et al., however, reported integration of a small amount of injected cells in the outer nuclear layer (ONL) of the retina, expression of photoreceptor markers, and the formation of outer segments [29]. Some improvement in light response was seen when they transplanted the cells in a mouse model of Leber's congenital amaurosis. Nevertheless, the small amount of integrated cells should be taken into account when interpreting these results.

While it has been shown that it is possible to generate photoreceptors from iPSCs with characteristics of developing photoreceptors, including membrane current, gene expression, and intermembrane channels [32], many challenges lie ahead in the quest to optimize a therapy. These issues include production of a highly homogenous population of donor photoreceptors from human iPSCs with inherent variabilities in the cell production process, manipulation of the virable host environment to allow optimized grafted cell survival and integration, and immune suppression. The groundwork for iPSC-based retinal therapy has already begun. Lamba's group reported injection of iPSC-derived photoreceptors in wildtype mice [33]. Three weeks after the subretinal transplantation, they found that a small amount of subretinally transplanted cells had migrated to the ONL, expressing photoreceptor-specific markers similar to those which were seen in human ESC-derived photoreceptors. Integration into the ONL, with some outer segment formation, was also observed in a degenerative swine model after transplantation of swine iPSC-derived rods [33]. Nevertheless, no improvement of retinal function was found using electroretinography, but this could be explained by the small amount of injected cells in comparison with the size of the swine retina. In any case, these studies offer guarded hope to individuals suffering from presently untreatable blinding diseases.

## 5. RGC Replacement Therapy and Optic Nerve Repair

Retinal ganglion cells are the major cell type affected in glaucoma and optic nerve injury conditions. Although there is substantial interest in RGC replacement, this approach likely faces the most formidable hurdles compared to the replacement of any other retinal cell types, considering that new RGCs must extend long nerve fibers, navigating through the optic nerve and chiasm into the brain and connecting with appropriate visual centers. RGC-like cells have been derived in vitro from mouse ESC-derived progenitors (Figure 3), as confirmed by the expression of RGC markers, such as Brn3b, Islet-1, and Thy-1 [34, 35]. Injection of ESC-derived RGC progenitors into the intravitreal cavity of rats showed migration and integration into different layers of the retina by a small number of cells. Mouse iPSCs have also been reported to be able to differentiate into RGC-like cells by overexpressing Math5 [36], which drove the expression of RGC markers similar to that seen in ESC-derived RGC-like cells but they showed limited integration in the mouse retina (Figure 3). In a different effort that aimed to protect and regenerate the optic nerve, noggin-induced neural progenitors derived from human iPSCs were injected into the rats with optic nerve crush injury [37]. In this model, some cells were seen integrating into the retina, and a higher number of surviving optic nerve axons as well as a significant increase in visual evoked potential were observed.

Recently, generation of three-dimensional (3D) self-organized optic vesicle-like structures with layered retinal neurons has been reported, using both mouse embryonic stem cells and human iPSCs [38, 39]. Over the course of 28 weeks, development of RGCs, horizontal cells, amacrine cells, Müller cells, bipolar cells, and light-responsive photoreceptors was observed. Despite the fact that optic nerve structure was not found in the ESC- or iPSC-derived eye cups, this culture system at least provides an enriched opportunity for studying the factors that control RGC development, pathology, and regeneration or repair. The ongoing developments in the iPSC field have taken many different forms that offer great prospects for disease modeling and the development of therapies for diseases that never had treatment options before.

## 6. Safety and Integrity of iPSC-Derived Cell Transplantation

Even though the inherent characteristic of iPSCs to divide indefinitely is particularly helpful in obtaining sufficient cells for transplantation, it could prove antagonistic by forming tumors through uncontrolled cell divisions. Elaborative research on the tumorigenicity of human stem cells in animal models is thus crucial before clinical studies can be started. Promising results were obtained by Kanemura et al., who detected no tumor growth after injection of suspensions of human iPSC-derived RPE cells into the subcutaneous tissue of immunodeficient mice [40]. Moreover, subretinal injection of human iPSC-derived RPE cell sheets also showed no tumor growth up to 15 months after transplantation. Accordingly, no tumor formation was seen in another study where RPE dystrophic rats were injected with iPSC-derived RPE cell suspensions subretinally [41]. The iPSC trial conducted by the Riken Institute, Japan, which focused on treating wet AMD with autologous iPSC-derived RPE cells, was recently put on hold due to the finding that the patient-specific iPSCs showed chromosomal abnormalities. Riken is now considering using a single iPSC line for the treatment of multiple patients in an allogeneic fashion [42]. Recent completion of two phase I/II clinical trials for subretinal transplantation of human ESC-derived RPE suspension in patients with AMD or Stargardt's diseases suggests no major adverse events or immune reactions over a medium follow-up of 22 months [43, 44]. Currently, clinical trials are ongoing for human ESC or iPSC-based RPEs for ocular diseases (Table 1). These studies provide a hope for stem cell-based therapies for possible visual improvement without tumor growth and/or other adverse effects.

At current efforts to develop an autologous cell replacement for AMD using iPSC-derived RPE, injection of cell suspensions is thought not to provide long-term survival or proper RPE functions. In one report, after initial formation of a cell layer, the transplanted cells were no longer detectable after 3 months [41]; nevertheless, an improved visual headtracking response was still observed in the transplanted eye, suggesting a possible protective effect on the host tissue. In another study, suspensionof iPSC-derived RPE was transplanted into mice with dysfunctional RPE, a model of retinitis pigmentosa [45]. Although only small number of cells was injected, improvement of function was seen in electroretinography tests, in the absence of tumor growth. In contrast, studies carried out with cell sheets derived from human iPSCs that were transplanted into a rod photoreceptor-dystrophic mouse model showed cell integration and development of cell-cell contacts [46]. In this strategy, RPE cells may be grown as a polarized monolayer on an artificial scaffold in vitro before transplantation. Currently, multiple approaches that utilize either no scaffold or an artificial scaffold to develop an RPE monolayer from iPSCs are being tested for increasing the survival and potential of cell replacement therapies for retinal degenerative diseases.

## 7. Conclusions and Future Application

Since Yamanaka first discovered that adult mouse fibroblasts could be induced to be reprogrammed into stem cells, much research has been done on the integrity, tumorigenicity, and therapeutic modalities of these cells. In the ophthalmic field, major steps have been taken especially toward therapies for neurodegenerative conditions. The increasingly improved quality of iPSC-derived RPE cells and photoreceptors has resulted in the first clinical trial in the ocular field, and the results are awaited with much interest. Also, encouraging results have been reported in deriving corneal epithelium cells, RGCs, and even 3D retinal structures from iPSCs. Key in these developments is that they occur without the involvement of human embryonic tissues, but adult somatic cells and several transcription factors. Targeted genomic editing or mutation repair (e.g., via CRISPR/Cas9, TALENs, and ZFNs) in harvested somatic cells, iPSCs, or iPSC-derived cells offers patients the opportunity to receive autologous cell therapy, while it also enables a broad range of research and medical applications. Cas9 nickase facilitates targeted DNA double-strand break using two guided RNAs so this versatile strategy enables a wide variety of genome editing applications that require high specificity [47]. Sluch et al. have reported a simple, adherent cell culture protocol for differentiation of human iPSCs into RGCs using a CRISPR-engineered RGC fluorescent reporter stem cell line. Fluorescence-activated cell sorting yields a highly purified population of cells that express a range of RGC-enriched markers and exhibit morphological and physiological properties typical of RGCs [48]. Currently, the full potential of iPSC-based therapy is yet to be realized because of the complexity and variations in reprogramming technology and protocols in driving specific ocular cell differentiation.

There are still important obstacles that must be considered. Despite the intensive research on iPSC-derived cell transplantation, the presence of remaining undifferentiated cells in the grafts poses a potential risk of tumor formation. Because the field is so young, long-term effects of reprogramming somatic cells are yet unknown. In the end, the technology of iPSCs is full of promise and offers modalities that were never before available to the medical field, including individualized disease modeling and personalized autologous grafts for transplantation purposes. More insight into the reprogramming process, its long-term effects, and the integrity of the generated cells would certainly be needed to bring iPSCs from the bench to bedside.

## Figures and Tables

**Table 1:** Clinical trials of ESC/iPSC-based studies in ocular therapy.

Cell type	Disease	Institute/location	Phase	Status of trial	References/sources
ESC-derived RPE	Dry AMD	Astellas Institute for Regenerative Medicine, USA	Phase I Phase II	Completed	NCT01344993
ESC-derived RPE	Stargardt's macular dystrophy	Astellas Institute for Regenerative Medicine, USA	Phase I Phase II	Completed	NCT01345006
ESC-derived RPE	Stargardt's macular dystrophy	Astellas Institute for Regenerative Medicine, UK	Phase I Phase II	Completed	NCT01469832
iPSC-derived RPE	Wet AMD	Rikagaku Kenkyûsho (RIKEN) Institute, Japan	Phase I	Halted	[42]
hESC-derived RPE	Wet AMD	Pfizer and University College, London, UK	Phase I	Ongoing	NCT01691261
hESC-derived RPE	Myopic macular degeneration	University of California and Ocata Therapeutics, USA	Phase I Phase II	Withdrawn	NCT02122159
hESC-derived RPE	Dry AMD	Cell Cure Neurosciences Ltd., Israel	Phase I Phase II	Recruiting	NCT02286089

AMD, age-related macular degeneration; hESC, human embryonic stem cell; iPSC, induced pluripotent stem cell; MMD, myopic macular degeneration; RPE, retinal pigment epithelium.

## References

[1] Klimanskaya I., Hipp J., Rezai K. A., West M., Atala A., Lanza R. (2004). Derivation and comparative assessment of retinal pigment epithelium from human embryonic stem cells using transcriptomics. *Cloning and Stem Cells*.

[2] Lu B., Malcuit C., Wang S. (2009). Long-term safety and function of RPE from human embryonic stem cells in preclinical models of macular degeneration. *Stem Cells*.

[3] Takahashi K., Yamanaka S. (2006). Induction of pluripotent stem cells from mouse embryonic and adult fibroblast cultures by defined factors. *Cell*.

[4] Seki T., Yuasa S., Kusumoto D. (2014). Generation and characterization of functional cardiomyocytes derived from human T cell-derived induced pluripotent stem cells. *PLoS ONE*.

[5] Takebe T., Sekine K., Enomura M. (2013). Vascularized and functional human liver from an iPSC-derived organ bud transplant. *Nature*.

[6] Phillips M. D., Kuznetsov S. A., Cherman N. (2014). Directed differentiation of human induced pluripotent stem cells toward bone and cartilage: in vitro versus in vivo assays. *Stem Cells Translational Medicine*.

[7] Miura K., Okada Y., Aoi T. (2009). Variation in the safety of induced pluripotent stem cell lines. *Nature Biotechnology*.

[8] Tsuji O., Miura K., Okada Y. (2010). Therapeutic potential of appropriately evaluated safe-induced pluripotent stem cells for spinal cord injury. *Proceedings of the National Academy of Sciences of the United States of America*.

[9] Shields T., Sloane P. D. (1991). A comparison of eye problems in primary care and ophthalmology practices. *Family Medicine*.

[10] Wipperman J. L., Dorsch J. N. (2013). Evaluation and management of corneal abrasions. *American Family Physician*.

[11] Cotsarelis G., Cheng S.-Z., Dong G., Sun T.-T., Lavker R. M. (1989). Existence of slow-cycling limbal epithelial basal cells that can be preferentially stimulated to proliferate: implications on epithelial stem cells. *Cell*.

[12] Dua H. S., Forrester J. V. (1990). The corneoscleral limbus in human corneal epithelial wound healing. *American Journal of Ophthalmology*.

[13] Frucht-Pery J., Siganos C. S., Solomon A., Scheman L., Brautbar C., Zauberman H. (1998). Limbal cell autograft transplantation for severe ocular surface disorders. *Graefe's Archive for Clinical and Experimental Ophthalmology*.

[14] Pellegrini G., Traverso C. E., Franzi A. T., Zingirian M., Cancedda R., De Luca M. (1997). Long-term restoration of damaged corneal surfaces with autologous cultivated corneal epithelium. *The Lancet*.

[15] Homma R., Yoshikawa H., Takeno M. (2004). Induction of epithelial progenitors in vitro from mouse embryonic stem cells and application for reconstruction of damaged cornea in mice. *Investigative Ophthalmology & Visual Science*.

[16] Hayashi R., Ishikawa Y., Ito M. (2012). Generation of corneal epithelial cells from induced pluripotent stem cells derived from human dermal fibroblast and corneal limbal epithelium. *PLoS ONE*.

[17] Mikhailova A., Ilmarinen T., Uusitalo H., Skottman H. (2014). Small-molecule induction promotes corneal epithelial cell differentiation from human induced pluripotent stem cells. *Stem Cell Reports*.

[18] Valerio L., Sugaya K. (2014). Potential therapeutic approaches for stroke using induced pluripotent stem cells. *Austin Journal of Biomedical Engineering*.

[19] Pennington B. O., Clegg D. O. (2016). Pluripotent stem cell-based therapies in combination with substrate for the treatment of age-related macular degeneration. *Journal of Ocular Pharmacology and Therapeutics*.

[20] Ferrari S., Di Iorio E., Barbaro V., Ponzin D., Sorrentino F. S., Parmeggiani F. (2011). Retinitis pigmentosa: genes and disease mechanisms. *Current Genomics*.

[21] Lopez R., Gouras P., Kjeldbye H. (1989). Transplanted retinal pigment epithelium modifies the retinal degeneration in the RCS rat. *Investigative Ophthalmology & Visual Science*.

[22] Hirami Y., Osakada F., Takahashi K. (2009). Generation of retinal cells from mouse and human induced pluripotent stem cells. *Neuroscience Letters*.

[23] Buchholz D. E., Hikita S. T., Rowland T. J. (2009). Derivation of functional retinal pigmented epithelium from induced pluripotent stem cells. *Stem Cells*.

[24] Kokkinaki M., Sahibzada N., Golestaneh N. (2011). Human induced pluripotent stem-derived retinal pigment epithelium (RPE) cells exhibit ion transport, membrane potential, polarized vascular endothelial growth factor secretion, and gene expression pattern similar to native RPE. *Stem Cells*.

[25] Singh R., Phillips M. J., Kuai D. (2013). Functional analysis of serially expanded human iPS cell-derived RPE cultures. *Investigative Ophthalmology & Visual Science*.

[26] Liao J.-L., Yu J., Huang K. (2010). Molecular signature of primary retinal pigment epithelium and stem-cell-derived RPE cells. *Human Molecular Genetics*.

[27] Maruotti J., Wahlin K., Gorrell D., Bhutto I., Lutty G., Zack D. J. (2013). A simple and scalable process for the differentiation of retinal pigment epithelium from human pluripotent stem cells. *Stem Cells Translational Medicine*.

[28] MacLaren R. E., Pearson R. A., MacNeil A. (2006). Retinal repair by transplantation of photoreceptor precursors. *Nature*.

[29] Lamba D. A., Gust J., Reh T. A. (2009). Transplantation of human embryonic stem cell-derived photoreceptors restores some visual function in Crx-deficient mice. *Cell Stem Cell*.

[30] West E. L., Gonzalez-Cordero A., Hippert C. (2012). Defining the integration capacity of embryonic stem cell-derived photoreceptor precursors. *STEM CELLS*.

[31] Osakada F., Ikeda H., Mandai M. (2008). Toward the generation of rod and cone photoreceptors from mouse, monkey and human embryonic stem cells. *Nature Biotechnology*.

[32] Homma K., Okamoto S., Mandai M. (2013). Developing rods transplanted into the degenerating retina of Crx-knockout mice exhibit neural activity similar to native photoreceptors. *Stem Cells*.

[33] Lamba D. A., McUsic A., Hirata R. K., Wang P.-R., Russell D., Reh T. A. (2010). Generation, purification and transplantation of photoreceptors derived from human induced pluripotent stem cells. *PLoS ONE*.

[34] Maekawa Y., Onishi A., Matsushita K. (2016). Optimized culture system to induce neurite outgrowth from retinal ganglion cells in three-dimensional retinal aggregates differentiated from mouse and human embryonic stem cells. *Current Eye Research*.

[35] Nistor G., Seiler M. J., Yan F., Ferguson D., Keirstead H. S. (2010). Three-dimensional early retinal progenitor 3D tissue constructs derived from human embryonic stem cells. *Journal of Neuroscience Methods*.

[36] Chen M., Chen Q., Sun X. (2010). Generation of retinal ganglion-like cells from reprogrammed mouse fibroblasts. *Investigative Ophthalmology and Visual Science*.

[37] Satarian L., Javan M., Kiani S., Hajikaram M., Mirnajafi-Zadeh J., Baharvand H. (2013). Engrafted human induced pluripotent stem cell-derived anterior specified neural progenitors protect the rat crushed optic nerve. *PloS ONE*.

[38] Eiraku M., Takata N., Ishibashi H. (2011). Self-organizing optic-cup morphogenesis in three-dimensional culture. *Nature*.

[39] Zhong X., Gutierrez C., Xue T. (2014). Generation of three-dimensional retinal tissue with functional photoreceptors from human iPSCs. *Nature Communications*.

[40] Kanemura H., Go M. J., Shikamura M. (2014). Tumorigenicity studies of induced pluripotent stem cell (iPSC)-derived retinal pigment epithelium (RPE) for the treatment of age-related macular degeneration. *PLoS ONE*.

[41] Carr A.-J., Vugler A. A., Hikita S. T. (2009). Protective effects of human iPS-derived retinal pigment epithelium cell transplantation in the retinal dystrophic rat. *PLoS ONE*.

[42] Reardon S., Cyranoski D. (2014). Japan stem-cell trial stirs envy. *Nature*.

[43] Schwartz S. G., Hickey M., Flynn H. W. (2013). Congenital hypertrophy of the retinal pigment epithelium: choroidal cavitation demonstrated on spectral-domain OCT. *Ophthalmic Surgery Lasers and Imaging Retina*.

[44] Schwartz S. D., Regillo C. D., Lam B. L. (2015). Human embryonic stem cell-derived retinal pigment epithelium in patients with age-related macular degeneration and Stargardt's macular dystrophy: follow-up of two open-label phase 1/2 studies. *The Lancet*.

[45] Li Y., Tsai Y.-T., Hsu C.-W. (2012). Long-term safety and efficacy of human-induced pluripotent stem cell (iPS) grafts in a preclinical model of retinitis pigmentosa. *Molecular Medicine*.

[46] Assawachananont J., Mandai M., Okamoto S. (2014). Transplantation of embryonic and induced pluripotent stem cell-derived 3D retinal sheets into retinal degenerative mice. *Stem Cell Reports*.

[47] Ran F. A., Hsu P. D., Lin C.-Y. (2013). Double nicking by RNA-guided CRISPR cas9 for enhanced genome editing specificity. *Cell*.

[48] Sluch V. M., Davis C.-H. O., Ranganathan V. (2015). Differentiation of human ESCs to retinal ganglion cells using a CRISPR engineered reporter cell line. *Scientific Reports*.

